# Sex-Dependent Effects of Eicosapentaenoic Acid on Hepatic Steatosis in UCP1 Knockout Mice

**DOI:** 10.3390/biomedicines9111549

**Published:** 2021-10-27

**Authors:** Kembra Albracht-Schulte, Savanna Wilson, Paige Johnson, Mandana Pahlavani, Latha Ramalingam, Bimba Goonapienuwala, Nishan S. Kalupahana, William T. Festuccia, Shane Scoggin, Chanaka N. Kahathuduwa, Naima Moustaid-Moussa

**Affiliations:** 1 Department of Nutritional Sciences, Obesity Research Institute, Texas Tech University, Lubbock, TX 79409, USA; kembra.albracht@ttu.edu (K.A.-S.); savannam355@gmail.com (S.W.); paige.n.johnson22@gmail.com (P.J.); mandana.pahlavani@utsouthwestern.edu (M.P.); lramalin@syr.edu (L.R.); bimba.goonapienuwala@ttu.edu (B.G.); skalupahana@pdn.ac.lk (N.S.K.); shane.scoggin@ttu.edu (S.S.); 2 Department of Physiology, Faculty of Medicine, University of Peradeniya, Peradeniya 20400, Sri Lanka; 3 Department of Physiology, Institute of Biomedical Sciences, University of São Paulo, São Paulo 05508-000, Brazil; william.festuccia@usp.br; 4 Texas Tech University Health Sciences Center, Department of Laboratory Sciences and Primary Care, Lubbock, TX 79430, USA; chanaka.kahathuduwa@ttuhsc.edu

**Keywords:** eicosapentaenoic acid (EPA), nonalcoholic fatty liver disease (NAFLD), obesity, omega-3 polyunsaturated fatty acids, thermoneutrality, uncoupling protein 1 (UCP1)

## Abstract

Visceral obesity may be a driving factor in nonalcoholic fatty liver disease (NAFLD) development. Previous studies have shown that the omega-3 polyunsaturated fatty acid, eicosapentaenoic acid (EPA), ameliorates obesity in high-fat (HF) fed male, C57Bl/6 mice at thermoneutral conditions, independent of uncoupling protein 1 (UCP1). Our goals herein were to investigate sex-dependent mechanisms of EPA in the livers of wild type (WT) and UCP1 knockout (KO) male and female mice fed a HF diet (45% kcal fat; WT-HF, KO-HF) with or without supplementation of 36 g/kg EPA (WT-EPA, KO-EPA). KO significantly increased body weight in males, with no significant reductions with EPA in the WT or KO groups. In females, there were no significant differences in body weight among KO groups and no effects of EPA. In males, liver TGs were significantly higher in the KO-HF group and reduced with EPA, which was not observed in females. Accordingly, gene and protein markers of mitochondrial oxidation, peroxisomal biogenesis and oxidation, as well as metabolic futile cycles were sex-dependently impacted by KO and EPA supplementation. These findings suggest a genotypic difference in response to dietary EPA supplementation on the livers of male and female mice with diet-induced obesity and housed at thermoneutrality.

## 1. Introduction

Visceral obesity, a disease characterized by increased fat accumulation in visceral adipose tissue, is a major risk factor for metabolic disease development, including nonalcoholic fatty liver disease (NAFLD) [1]. Specifically, white adipose tissue (WAT) is a key contributor to NAFLD pathophysiology since enlarged WAT instigates inflammation, insulin resistance, and enhanced lipolysis, which increases fatty acid flow to the liver [2,3]. Excessive hepatocyte triglyceride (TG) accumulation prompts NAFLD development and progression to nonalcoholic steatohepatitis (NASH), which is characterized by inflammation, fibrosis, and collagen deposition [2]. NAFLD is a major contributor to chronic liver disease with an estimated global prevalence of 24% [4] and a prevalence of 30% in the United States [5].

In contrast to the role of WAT in NAFLD development, brown adipose tissue (BAT) oxidizes fatty acids and glucose to produce heat [6], and thus, may consequently avert hepatic steatosis. Indeed, few studies have reported that BAT activation limits hepatic lipid accumulation and damage [7]. Mitochondrial uncoupling proteins (UCPs), primarily uncoupling protein 1 (UCP1), act in uncoupling the electron transport chain and ATP synthesis, thus increasing the dissipation of energy as heat instead of ATP, in a process known as non-shivering thermogenesis [8]. Utilizing transgenic animals, several studies have demonstrated the anti-obesity and insulin sensitizing effects in adipocytes [9] and skeletal muscle [10] with UCP1 overexpression. Conversely, UCP1 knockout (KO) mice have an increased susceptibility to the adverse effects of a high fat (HF) diet and are prone to glucose intolerance and hepatic steatosis [11,12]. Therefore, activation of UCP1 may be a promising therapeutic strategy to counteract obesity and NAFLD.

Lipid storage and metabolism, fat distribution, as well as the metabolic consequences associated with obesity vary by sex [13,14]. Despite these differences, most obesity related studies utilize only male mice since they are more prone to diet-induced metabolic dysfunction compared to females [15,16]. However, therapies that target molecular processes involved in regulating energy balance should be evaluated in both males and females. Nutritional bioactive compounds that target these energy-regulating proteins are of interest as prevention/treatment options for obesity-related diseases, such as NAFLD [17].

Dietary omega-3 (n-3) polyunsaturated fatty acids (PUFA), such as eicosapentaenoic acid (EPA; C20:5n-3), commonly found in fish oil, are a promising anti-obesity bioactive with potential in improving NAFLD [18,19]. Metabolism of n-3 PUFAs may be sex-specific [20,21], and therefore n-3 PUFAs may have differing implications in the treatment of obesity-associated diseases, including NAFLD, in males and females. Utilizing HF diet-induced male, obese B6 mice housed at 22 °C, we reported that EPA supplementation (36 g EPA/kg of diet; 6.75% kcal fat) reduced hepatic TG accumulation [19], body weight, and adiposity [15], such an effect that was associated with increased expression of UCP1 in BAT, suggesting a possible activation of non-shivering thermogenesis [22]. To further test this, UCP1 KO male and female mice were fed a similar diet; but since UCP1 KO mice only gain weight at thermoneutrality [6], both wild type (WT) and UCP1 KO mice were housed in a thermoneutral environment (28–30 °C). Surprisingly, EPA remained effective in reducing body weight and fat mass and attenuating glucose intolerance in the KO model (assessed by two-way ANOVA and without statistical consideration of sex), indicating that the effects of EPA are independent of UCP1 in both males [16] and females [23]. Moreover, oxygen consumption was increased with EPA in the male KO mice [16] and female WT mice, with no significant differences observed between the HF and HF-EPA female KO mice [24].

Liver is a major organ in regulating lipid metabolism and glucose homeostasis; however, hepatic effects of EPA in HF diet-fed UCP1 KO mice have not been considered in males and females. In this study, we investigated the protective effects of EPA in attenuating hepatic steatosis induced by HF diet and UCP1 KO by assessing mitochondrial and peroxisomal hepatic lipid oxidation as well as mitochondrial metabolic futile cycling. Further, we compared the influence of these pathways in male and female mice.

## 2. Materials and Methods

### 2.1. Animal Studies

Genotyping for the UCP1 KO mice, experimental groups, and study design have been previously described [16,23]. Briefly, heterozygous UCP1 C57BL/6J male and female mice (Jackson Laboratory, Bar Harbor, ME, USA) were bred to produce homozygous UCP1 KO and WT groups. Mice from both WT and UCP1 KO groups were housed at 22 °C from birth until aged 5–6 weeks and were then randomized (10–12 mice per group; 80–96 total) to receive either a HF diet (45% fat, 20% protein and 35% carbohydrate) or a HF diet supplemented with EPA-enriched fish oil (36 g/kg) for 14 weeks. AlaskOmega EPA-enriched fish oil was provided, in kind, by Organic Technologies (Coshocton, OH, USA) and incorporated into mouse diets by Research Diets, Inc (New Brunswick, NJ, USA). Detailed diet information is provided in Supplementary Table S1 [16]. Mice were housed at thermoneutral temperature (28–30 °C). Food intake was recorded twice per week. At the end of the 14 weeks, mice were euthanized utilizing the CO_2_ inhalation method following 5 h of feed deprivation. Livers of these mice were collected and used for analyses. The Institutional Animal Care and Use Committee (IACUC) of Texas Tech University (TTU-16011-04) approved all procedures.

### 2.2. Liver Fatty Acid Composition

As previously described, direct fatty acid methyl ester (FAME) synthesis and gas chromatography/mass spectrometry methods were utilized to identify liver fatty acid (FA) concentrations (total lipid) and to validate EPA delivery to the liver in the WT-EPA and KO-EPA mice [25,26]. All groups and both male and female liver samples were analyzed.

### 2.3. Liver Histology

Liver sections were fixed in 10% buffered formalin, routinely processed, sectioned at 5 mm, and stained with hematoxylin and eosin (H & E). Sections were imaged at 20× magnification.

### 2.4. Liver Triglycerides

Triglyceride Colorimetric Assay Kit (Cayman Chemical Company, Ann Arbor, MI, USA) was used to determine triglyceride content in liver. Tissue homogenates were prepared according to the manufacturer procedures. Triglyceride concentrations were normalized to protein concentrations.

### 2.5. RNA Isolation and Quantitative Real-Time PCR

Total RNA from liver was extracted using the Qiagen RNeasy kit (Qiagen, Valencia, CA, USA). cDNA synthesis was performed using the Maxima First Strand cDNA Synthesis Kit (Thermo Fisher Scientific Inc., Waltham, MA, USA). Using Syber Green PCR Master Mix (Bio-Rad, Hercules, CA, USA), real-time quantitative PCR (RT-PCR) was conducted (Bio-Rad) to assess gene expression, normalized to the expression of glyceraldehyde 3-phosphate dehydrogenase (*Gapdh*) and *18s* and calculated utilizing the ΔΔCt method. Primers were designed using OligoArchitect™ Online and purchased from Sigma-Aldrich (St. Louis, MO, USA). Primer sequences are provided in Supplementary Table S2.

### 2.6. Immunoblotting

Proteins were extracted from livers by homogenizing in modified radio-immunoprecipitation (RIPA) assay buffer (Thermo Fisher Scientific Inc.). Protein was loaded in equal amounts per lane and separated using TGX Stain-Free™ FastCast™ Gels (Bio-Rad) of appropriate gradients and transferred to a polyvinylidene fluoride (PVDF) membrane using Immobilon-FL Transfer Membranes (MilliporeSigma, Burlington, MA, USA). The PVDF membrane was blocked using Blocker™ FL Blocking Buffer (Thermo Fisher Scientific Inc.) for an hour followed by incubation with primary antibodies for fatty acid synthase (FASN) (Santa Cruz Biotechnology, CA, USA; dilution 1:1000) and phosphorylated (Thr172) [27] and total AMP-activated protein kinase (AMPK) (Cell Signaling Technologies, Danvers, MA, USA; dilution 1:500). Protein concentrations were normalized to TATA-Box binding protein (TBP; Cell Signaling Technologies; dilution 1:1000). Mouse polyclonal antibody was used as a secondary antibody for FASN (dilution 1:25,000), and rabbit polyclonal antibody was used as a secondary antibody for AMPK (dilution 1:25,000).

### 2.7. Statistical Analyses

Results are presented as means ± standard error of the mean (SEM). Using Prism (GraphPad Software 9.2.0, San Diego, CA, USA), data were analyzed by performing three-way analysis of variance (ANOVA), including a main effect for genotype (WT and UCP1 KO), diet (HF and HF-EPA), and sex (male and female) and their interactions. If significant, Tukey-corrected post-hoc pairwise comparisons were made maintaining Family-wise error rate at 0.05.

## 3. Results

### 3.1. UCP1 Deficiency Sex-dependently Affects Body Weight

**Figure 1:**
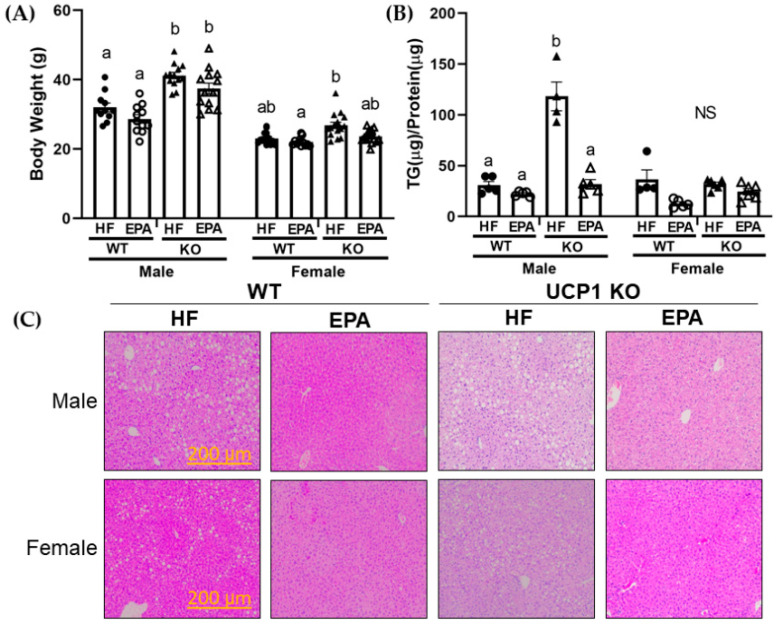
EPA reduces hepatic triglycerides (TG) in UCP1 KO males independent of body weight. (**A**) Final body weights of male and female wild type (WT) and UCP1 knockout (KO) mice. UCP1 KO significantly increased body weight in males but had no impact in female mice. There was no significant impact of EPA on body weight in either WT or KO male or female mice groups. Data are expressed as mean ± SEM, *n* = 13–15, means without a common letter (a,b) differ in each group of males and females; groups with any common letter are not different from each other (a or b vs. ab). NS indicates no significant post-hoc comparisons (**B**) Hepatic TG content in male and female WT-HF, WT-EPA, KO-HF and KO-EPA groups. UCP1 KO significantly increased hepatic TGs in males, which was significantly reduced with EPA. Data are expressed as mean ± SEM, *n* = 4–6, means without a common letter (a, b) differ in each male or female group; means with any common letter are not different from each other (a or b vs. ab). NS indicates no significant post-hoc comparisons. (**C**) Representative hematoxylin and eosin-stained sections of the liver from WT-HF, WT-EPA, KO-HF and KO-EPA groups are shown for both male and female mice.

In a thermoneutral environment, UCP1 KO mice develop HF diet-induced obesity [28]. Moreover, data from our lab and others indicate anti-obesity effects with EPA supplementation to HF feeding in male mice housed in ambient temperature (22–25 °C) [15,18,29]. However, given the association between obesity and NAFLD, and lack of data in female mice [30], we evaluated the influence of EPA supplementation on body weight in the WT and UCP1 KO groups in both male and female mice. The three-way ANOVA revealed significant main effects for genotype (F (1, 96) = 73.57, *p* < 0.0001), diet (F (1, 96) = 17.14, *p* < 0.0001) and sex (F (1, 96) = 259.3, *p* < 0.0001). There was also a significant sex x genotype interaction (F (1, 96) = 21.58, *p* < 0.0001) (Table 1). In the males, post-hoc comparisons indicate KO groups weighed significantly greater than WT groups at sacrifice with no significant differences in food intake (previously reported by Pahlavani et al.) and no differences between diet groups within the same genotype [16]. In the females, post-hoc comparisons demonstrated no relevant impact of EPA among the WT or KO groups, with no significant differences between the HF and EPA fed mice in either genotype. Female mice in the WT-EPA group weighed significantly less than the KO-HF group (Figure 1A).

### 3.2. Dietary EPA Increases Hepatic EPA Content

Fatty acid analysis of liver tissue from all groups revealed significant hepatic enrichment of EPA in both the male and female groups with dietary EPA (Table 2). The three-way ANOVA revealed a significant main effect for diet (F (1, 20) = 329.5, *p* < 0.0001). No significant main effects were found for sex or genotype, and none of the interactions were significant. For both males and females, post-hoc comparisons indicate significant increases in hepatic EPA content in the EPA fed groups compared to the HF fed groups. Regarding hepatic content of other n-3 PUFAs, including docosahexaenoic acid (DHA), the three-way ANOVA also revealed a significant main effect for diet (F (1, 20) = 116.6, *p* <0.0001). There were no significant main effects for sex or genotype, and none of the interactions were significant. In males, post-hoc comparisons indicate significant increases in hepatic DHA content with EPA supplementation; however, for females, this increase was only seen in the KO groups.

Arachidonic acid (AA) was reduced (trending) in EPA-fed groups [19]. The three-way ANOVA revealed a significant main effect for genotype and diet for AA (genotype: F (1, 20) = 5.42, *p* = 0.03; diet: F (1, 20) = 22.77, *p* = 0.0001) (Table 2). In males, post-hoc comparisons indicate significant increases in the WT-HF group compared to the KO-EPA group. There were no significant differences among groups in the females. Interestingly, the three-way ANOVA revealed a significant main effect for sex for both linoleic acid (n-6 PUFA; F (1, 20) = 1012, *p* < 0.0001) and linolenic acid (n-3 PUFA; F (1, 20) = 147.4, *p* < 0.0001), with no significant differences among male or female groups revealed by post-hoc comparisons (Table 2). However, females had much lower hepatic linoleic levels compared to males. Additionally, the three-way ANOVA revealed a significant main effect for sex and diet for palmitoleic (sex: F (1, 20) = 4.85, *p* = 0.04, diet: F (1, 20) = 11.79, *p* = 0.003) and palmitic acid (sex: F (1, 20) = 76.78, *p* < 0.0001, diet: F (1, 20) = 0.232, *p* = 0.008). In males, post-hoc comparisons indicate significant decreases in the WT-EPA group compared to the WT-HF and KO-HF group for palmitoleic acid and no significant differences among groups for palmitic acid content. In females, post-hoc comparisons indicate no differences among groups for palmitoleic acid. Interestingly, female EPA groups had significantly higher hepatic palmitic acid content.

### 3.3. EPA Reduces Hepatic Triglycerides

The three-way ANOVA for liver TG normalized to protein revealed significant main effects for sex (F (1, 32) = 40.96, *p* < 0.0001), genotype (F 1, 32) = 46.23, *p* < 0.0001), and diet (F (1, 32) = 68.59, *p* < 0.0001) as well as significant two-way interactions for sex × genotype (F (1, 32) = 35.23, *p* < 0.0001), sex × diet (F (1, 32) = 17.27, *p* = 0.0002), and genotype x diet (F (1, 32) = 16.01, *p* = 0.0003) as well as the three-way interaction, sex × genotype × diet (F (1, 32) = 38.00, *p* < 0.0001; Table 1). In males, UCP-1 knockdown led to increased liver TG content, which was rescued by EPA supplementation. It is important to note that this EPA effect was independent of body weight, since there was no difference in body weight between HF and EPA fed male UCP1-KO mice. However, there was no significant difference in the WT-HF and WT-EPA groups. No significant post-hoc comparisons were noted among the female groups (Figure 1B), despite visual observations in the H&E-stained sections between the HF and EPA fed female groups. H&E staining of liver was used to visually assess TG accumulation through light-microscopy (Figure 1C).

### 3.4. EPA Rescues Hepatic Triglyceride Load by Upregulating Fat Oxidation

Given the differences in hepatic TG accumulation, we next examined the effects of EPA and UCP1 KO on markers of hepatic lipid metabolism. To determine these effects, we used livers from WT and UCP1 KO mice fed HF or HF diets supplemented with EPA. AMPK, a regulator of lipid metabolism, as well as protein and gene expression of known contributors to FA synthesis and TG packaging, including acetyl-coA carboxylase (Acaca), fatty acid synthase (Fasn), and diacylglycerol O-acyltransferase 2 (Dgat2), were assessed.

**Figure 2:**
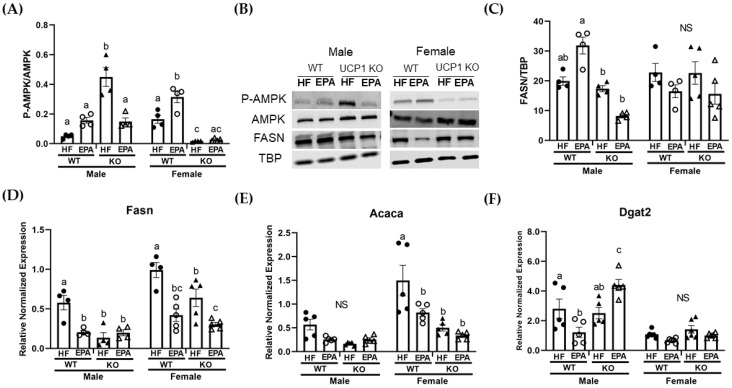
Sex-dependent effects on markers of hepatic lipogenesis. (**A**) Protein quantification of P-AMPK normalized to total-AMPK. In males, P-AMPK was significantly increased in the KO-HF group compared to all other groups, which were comparable. In females, P-AMPK was significantly increased with EPA in the WT groups. Protein levels were comparable between female KO groups. (**B**) Representative western blot images in male and female groups. (**C**) Fatty acid synthase (FASN), normalized to TBP in male and female groups. Gene expression of lipogenic markers in male and female groups, including (**D**) *Fasn*, (**E**) acetyl-coA carboxylase (*Acaca*), and (**F**) diacylglycerol O-acyltransferase 2 (*Dgat2*). (**A**–**F**) Data are expressed as mean ± SEM, *n* = 4–6; means without a common letter (a, b, c) differ in each male or female group; means with any common letter are not different from each other (a or b vs. ab; b or c vs. bc; or b vs. bc or ab). NS indicates no significant post-hoc comparisons.

Of the proteins assessed, the three-way ANOVA revealed a significant main effect for sex (F (1, 24) = 10.90, *p* = 0.003) for phosphorylated AMPK/total AMPK. Additionally, there were significant sex x genotype (F (1, 24) = 93.56, *p* < 0.0001), sex × diet (F (1, 24) = 16.98, *p* = 0.0004) and genotype × diet (F (1, 24) = 40.96, *p* < 0.0001) interactions. The three-way ANOVA also revealed a significant sex × genotype × diet interaction (F (1, 24) = 9.78, *p* = 0.005; Table 1). In males, post-hoc comparisons indicate trending increases with EPA in the WT group with significant increases in the KO-HF group compared to all other groups. In females, P-AMPK was significantly increased with EPA in the WT groups. Additionally, protein levels were comparable between female KO groups (Figure 2A,B). For FASN, the three-way ANOVA revealed a significant main effect for genotype (F (1, 26) = 12.30, *p* = 0.002) as well as significant sex × genotype (F (1, 26) = 10.47, *p* = 0.003), sex x diet (F (1, 26) = 4.27, *p* = 0.048), and genotype × diet (F (1, 26) = 7.85, *p* = 0.01) interactions. Additionally, there was a significant sex × genotype × diet interaction (F (1, 26) = 6.88, *p* = 0.01); Table 1). In males, post-hoc analyses indicate no significant differences among the WT groups or the KO groups. In females, there were no significant differences among the groups (Figure 2B,C).

The three-way ANOVA revealed a significant main effect for genotype for all three markers of lipogenesis, including Acaca (F (1, 32) = 27.18, *p* = 0.0001), Fasn (F (1, 27) = 18.86, *p* = 0.0002) and Dgat2 (F (1, 36) = 16.42, *p* = 0.0003), and a main effect for diet for Acaca (F (1, 32) = 8.59, *p* = 0.006) and Fasn (F (1, 27) = 33.21, *p* < 0.0001). There was no significant main effect for diet for Dgat2. The three-way ANOVA also revealed a significant main effect for sex for all three markers of lipogenesis, including Acaca (F (1,32) = 27.9, *p* <0.0001), Fasn (F (1, 27) = 34.25, *p* <0.0001) and Dgat2 (F (1, 36) = 58.66, *p* <0.0001). A significant sex × genotype interaction was observed for Acaca (F (1, 32) = 8.87, *p* = 0.006) and Dgat2 (F (1, 36) = 6.30, *p* = 0.02) as well as a significant sex × diet interaction for both Acaca (F (1, 32) = 2.88, *p* = 0.01) and Fasn (F (1, 27) = 7.94, *p* = 0.009). Additionally, a significant genotype × diet interaction was observed for all three markers of lipogenesis, including Acaca (F (1, 32) = 6.41, *p* = 0.02), Fasn (F (1, 27) = 10.02, *p* = 0.004) and Dgat2 (F (1, 36) = 15.55, *p* = 0.0004). No significant sex × genotype × diet interaction was observed for Acaca or Fasn; however, a significant sex × genotype × diet interaction was observed for Dgat2 (F (1, 36) = 15.41, *p* = 0.0004). Despite significant differences in TG between the KO-HF and KO-EPA groups in males, there were no significant differences between these male groups for Fasn (Figure 2D) or Acaca (Figure 2E). However, Fasn was significantly reduced with EPA in the WT male group. By contrast, post-hoc comparisons indicate that there was increased expression of Dgat2 in the KO-EPA male group (Figure 2F). In females, post-hoc comparisons indicate Fasn and Acaca were significantly reduced in the KO-HF group compared to the WT-HF group and with EPA in the WT groups. Only Fasn was significantly decreased with EPA in the KO female group (Figure 2D).

Since hepatic TG results did not parallel changes in markers of lipogenesis, particularly in the male group, and our lab has previously demonstrated that EPA increased mitochondrial beta-oxidation in the livers of HF fed mice [19], we then examined markers of both mitochondrial and peroxisomal oxidation. Given the inactivation of UCP1 in our KO group, we assessed the mRNA levels of other uncoupling proteins which may be more highly expressed in the liver, including UCP2 and UCP3, and which may be involved in lipid handling [27,31,32,33].

**Figure 3:**
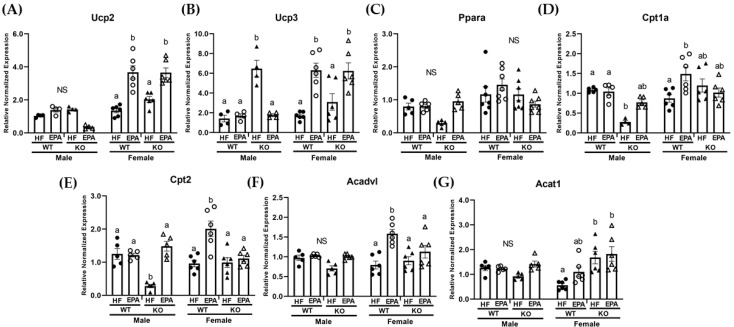
Sex-dependent effects on markers of hepatic lipid handling and fat oxidation. Gene expression of uncoupling proteins (Ucp) mRNA level of genes, (**A**) *Ucp2* and (**B**) *Ucp3* as well as genes related to lipid handling and mitochondrial oxidation, including (**C**) peroxisome proliferator-activated receptor-alpha (*Ppar-α*), (**D**) carnitine palmitoyltransferase (*Cpt*)*1a*, (**E**) *Cpt2*, (**F**) very long-chain specific acyl-CoA dehydrogenase (*Acadvl*), and (**G**) acetyl-CoA acetyltransferase (*Acat1*) in male and female WT and UCP1 KO groups fed HF diets and EPA supplemented HF diets. (**A**–**G**) Data are expressed as mean ± SEM, *n* = 4–6; means without a common letter (a, b) differ in each male or female group; means with any common letter (a or b vs. ab) are not different from each other. NS indicates no significant post-hoc comparisons.

The three-way ANOVA revealed a significant main effect for genotype for Ucp3 (F (1, 32) = 11.77, *p* = 0.002) and a main effect for diet for Ucp2 (F (1, 32) = 21.99, *p* < 0.0001) as well as a significant main effect for sex for both Ucp2 (F (1, 32) = 89.90, *p* < 0.0001) and Ucp3 (F (1, 32) = 10.19, *p* = 0.003). There was also significant sex × diet interaction (Ucp2: (F (1, 32) = 45.87, *p* < 0.0001; Ucp3: F (1, 32) = 42.02, *p* < 0.0001) and genotype × diet interaction for both Ucp2 (F (1, 32) = 9.57, *p* = 0.004) and Ucp3 (F (1, 32) = 11.79, *p* = 0.002). A significant sex × genotype × diet interaction was not observed for either Ucp2 or Ucp3 (Table 1). In males, post-hoc comparisons indicated no significant difference among groups for Ucp2 (Figure 3A), but a significant increase in the expression of Ucp3 (Figure 3B) in the KO-HF group compared to all other groups. In females, post-hoc comparisons indicated no significant differences between WT-HF and KO-HF groups and significant increase in both Ucp2 and Ucp3 in the EPA fed WT and KO groups compared to their respective HF groups (Figure 3A,B).

Next, gene expression of known key mitochondrial oxidation markers, including the transcriptional factor, peroxisome proliferator-activated receptor-alpha (Ppar-α), carnitine palmitoyltransferase (Cpt)1a, Cpt2, very long-chain specific acyl-CoA dehydrogenase (Acadvl), and acetyl-CoA acetyltransferase (Acat1) were assessed. The three-way ANOVA revealed a significant main effect for genotype for Ppar-α (F (1, 40) = 4.48, *p* = 0.04), Cpt1a (F (1, 36) = 12.62, *p* = 0.001), Cpt2 (F (1, 36) = 15.40, *p* = 0.0004), Acadvl (F, (1, 36) = 5.09, *p* = 0.03), and Acat1 (F (1, 36) = 10.65, *p* = 0.002). There was also a significant main effect for diet for Cpt1a (F (1, 36) = 6.79, *p* = 0.01), Cpt2 (F (1, 36) = 33.86, *p* < 0.0001), Acadvl (F (1, 36) = 23.89, *p* < 0.0001), and Acat1 (F (1, 36) = 5.01, *p* = 0.03). Additionally, the three-way ANOVA revealed a significant main effect for sex for Ppar-α (F (1, 40) = 15.99, *p* = 0.0003), Cpt1a (F (1, 36) = 16.67. *p* = 0.0002), Cpt2 (F (1, 36) = 4.41, *p* = 0.04) and Acadvl (F (1, 36) = 5.95, *p* = 0.02). There was also a significant sex × genotype interaction for Cpt1a (F (1, 36) = 7.40, *p* = 0.01) and Acat1 (F (1, 36) = 14.90, *p* < 0.0001). There were no significant genotype × diet interactions and only a significant sex x diet interaction for Acadvl (F (1, 36) = 5.50, *p* = 0.03). Additionally, the three-way ANOVA revealed a significant sex × genotype × diet interaction for Ppar-α (F (1, 36) = 7.56, *p* = 0.009), Cpt1a (F (1, 36) = 15.10, *p* = 0.0004), Cpt2 (F (1, 36) = 29.17, *p* < 0.0001) and Acadvl (F (1, 36) = 8.17, *p* = 0.007) (Table 1). In males, post-hoc comparisons indicate no significant differences in Ppar-α (Figure 3C). Both Cpt1a (Figure 3D) and Cpt2 (Figure 3E) were significantly reduced in the KO-HF male group compared to the WT-HF group, with a significant recovery in Cpt2 expression with EPA supplementation in the KO group (Figure 3E). Expression of Acadvl and Acat1 were not significantly different among the groups (Figure 3F,G). In females, post-hoc comparisons indicated no significant differences in Ppar-α (Figure 3C) among groups and comparable Cpt1a, Cpt2 and Acadvl expression between WT-HF and KO groups that were significantly up-regulated with EPA in the WT group (Figure 3D–F). Compared to the WT-HF group, there was a significant increase in Acat1 expression in the KO-HF group and a trending significant up-regulation with EPA in the WT group with no significant differences among the KO groups (Figure 3G).

Lastly, expression of genes associated with peroxisomal biogenesis and β-oxidation including, enoyl-CoA hydratase (Ehhadh), peroxisomal biogenesis factor 5 (Pex5) and acox-acyl coA oxidase (Acox1) were assessed. The three-way ANOVA revealed a significant main effect for genotype for Ehhadh (F (1, 36) = 5.68, *p* = 0.03), and Acox1 (F (1, 36) = 6.87, *p* = 0.01). A significant main effect for diet was also observed for Ehhadh (F (1, 36) = 42.84, *p* < 0.0001) and Acox1 (F (1, 36) = 22.65, *p* < 0.0001). No significant main effect for sex was reported. No significant main effect for sex was reported. The three-way ANOVA also revealed a significant sex × genotype interaction for Ehhadh (F (1, 36) = 23.72, *p* < 0.0001), Pex5 (F (1, 36) = 6.58, *p* = 0.02), and Acox1 (F (1, 36), *p* = 0.008). There was also a significant sex × genotype × diet interaction for all three genes, including Ehhadh (F (1, 36) = 12.99, *p* = 0.0009), Pex5 (F (1, 36) = 7.52, *p* = 0.01), and Acox1 (F (1, 36) = 6.52, *p* = 0.02; Table 1).

**Figure 4:**
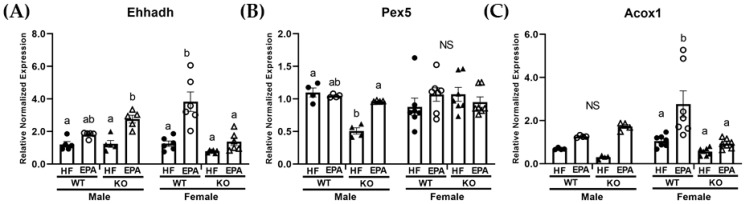
Sex-dependent effects on markers of hepatic peroxisomal fatty acid (FA) oxidation. mRNA level of (**A**) enoyl-CoA hydratase (*Ehhadh*), (**B**) peroxisomal biogenesis factor 5 (*Pex5*) and (**C**) acox-acyl coA oxidase (*Acox1*) in male and female WT and UCP1 KO groups fed HF diets and EPA supplemented HF diets. (**A**–**C**) Data are expressed as mean ± SEM, *n* = 4–6; means without a common letter (a, b) differ in each male or female group; means with any common letter (a or b vs. ab) are not different from each other. NS indicates no significant post-hoc comparisons.

In males, post-hoc comparisons indicated a significant decrease in Pex5 in the KO-HF group compared to other groups and significant increases in Ehhadh (Figure 4A) and Pex5 (Figure 4B) expression with EPA supplementation in the KO groups, with comparable findings among the WT groups. Post-hoc comparisons indicated no significant differences among groups in the expression of Acox1 (Figure 4C). In the females, post-hoc comparisons indicated that Ehhadh (Figure 4A) and Acox1 (Figure 4C) expression were comparable among WT-HF and KO-HF groups. Additionally, both were significantly upregulated with EPA in the WT groups with no significant differences in the KO groups. Pex5 was comparable among all female groups (Figure 4B).

### 3.5. EPA Alters Metabolic Futile Cycles

**Figure 5:**
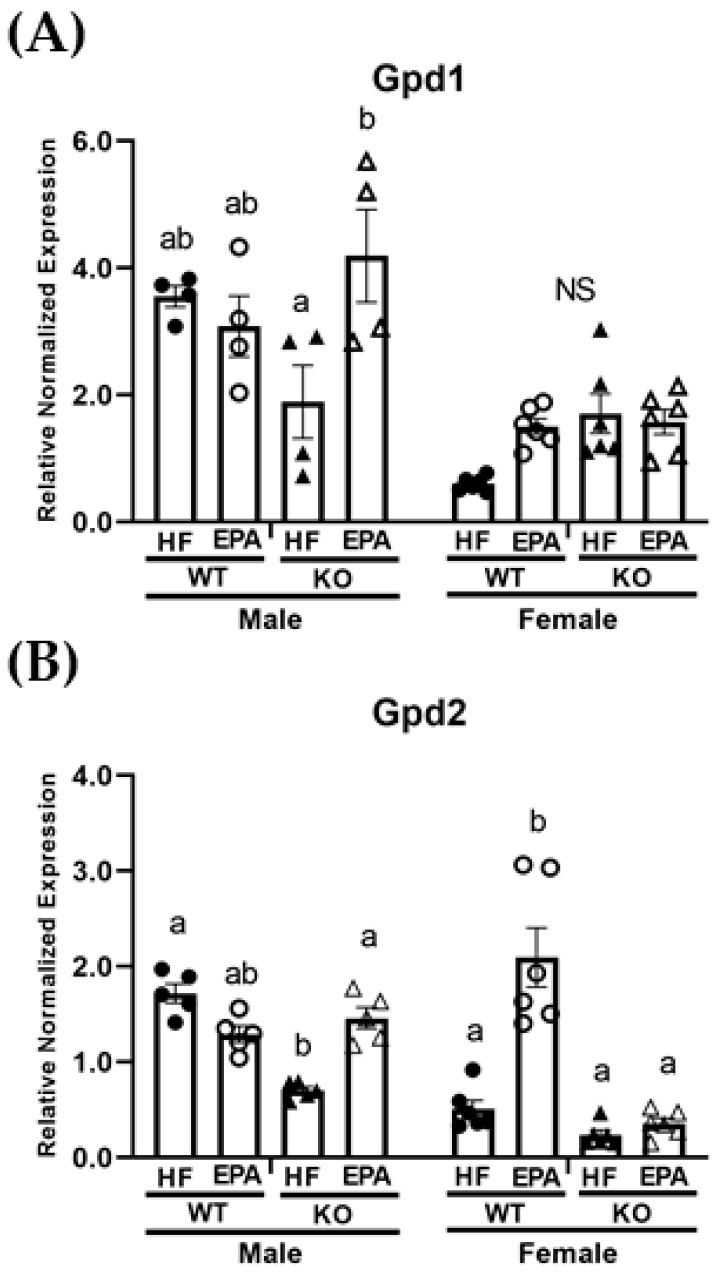
Sex-dependent effects on markers of metabolic futile cycling. mRNA levels of (**A**) *glycerol 3-phosphate dehydrogenase (Gpd) 1* and (**B**) *Gpd2* in the livers of male and female WT and UCP1 KO groups fed HF diets and EPA supplemented HF diets. (**A**,**B**) Data are expressed as mean ± SEM, *n* = 4–6; means without a common letter (a, b) differ in each male or female group; means with any common letter (a or b vs. ab) are not different from each other. NS indicates no significant post-hoc comparisons.

Additional thermogenic processes, such as metabolic futile cycling involving glycerol 3-phosphate catalyzed by glycerol 3-phosphate dehydrogenase (GPD) through the paired activity of mitochondrial GPD2 and cytosolic GPD1, may compensate in the absence of UCP1 [34]. Thus, we next assessed gene expression of Gpd1 and Gpd2 in the livers of UCP1 KO and WT mice fed HF and HF-EPA diets. The three-way ANOVA revealed significant main effect for genotype for Gpd2 (F (1, 36) = 51.07, *p* < 0.0001) and significant main effects for diet and sex for both Gpd1 (diet: F (1, 36) = 7.22, *p* = 0.01; sex: F (1, 36) = 57.29, *p* < 0.0001) and Gpd2 (diet: F (1, 25.93, *p* < 0.0001; sex: F (1, 36) = 24.40, *p* < 0.0001). The three-way ANOVA also revealed significant sex × genotype (F (1, 36) = 8.44, *p* = 0.006) and sex × diet (F (1, 36) = 11.88, *p* = 0.002) interactions for Gpd2. A significant sex × genotype × diet interaction for both Gpd1 (F (1, 36) = 15.42, *p* = 0.0004) and Gpd2 (F (1, 36) = 44.04, *p* < 0.0001) was also observed (Table 1). In males, post-hoc comparisons indicate comparable expression for Gpd1 (Figure 5A) among WT-HF and KO-HF groups and a significant reduction in Gpd2 (Figure 5B) in the KO-HF group compared to the WT-HF group. Additionally, significant increases in both Gpd1 and Gpd2 were noted with EPA supplementation in the KO group, with comparable expressions in the WT groups. In females, post-hoc comparisons indicated no significant differences in Gpd1 (Figure 5A). No significant differences in Gpd2 were observed among WT-HF and KO-HF groups, but there was a significant increase in Gpd2 expression with EPA supplementation in the WT group (Figure 5B).

## 4. Discussion

In the United States, approximately 35% of the adult population has obesity (defined as BMI > 30) [35] and the majority of these develop metabolic complications, including NAFLD [36], which is projected to be a disease of significant concern within the next 20 years [37]. Therefore, it is important to understand the role of BAT and related mitochondrial proteins as both (1) limited in obesity and a potential contributor to NAFLD pathogenesis and/or as (2) a target for therapeutic anti-obesity agents, both of which could have implications in the prevention and treatment of NAFLD.

In this study, we report a genotypic difference (WT vs. KO) in response to dietary EPA supplementation (36 g/kg) on the livers of male and female mice housed in a thermoneutral environment. For males, we report an increase in hepatic TG in response to UCP1 KO and HF feeding. However, under these same conditions, EPA significantly reduced hepatic TG accumulation in males, which may occur through: (1) upregulated mitochondrial and peroxisomal β-oxidation, and (2) activating mitochondrial metabolic futile cycles. For the females, we report no hepatic impact of UCP1 KO. Interestingly, EPA marginally reduced female hepatic TG accumulation in the WT group under thermoneutral conditions, which may occur through: (1) reduced FA synthesis, (2) activity of UCPs, (3) upregulated mitochondrial and peroxisomal β-oxidation and (4) activating mitochondrial metabolic futile cycles.

In agreement with our previous reports [19], dietary EPA supplementation is reflected in hepatic tissue, with significant increases in hepatic EPA concentrations in the WT-EPA and KO-EPA groups in both males and females. We also report significant increases in hepatic DHA concentrations, despite limited dietary DHA supplementation, which could indicate increased hepatic conversion of EPA to DHA. We did not find this in our previous study comparing HF and HF-EPA diet effects on liver conducted at 22 °C [19], indicating that hepatic n-3 PUFA composition and conversion may be impacted by environmental temperature [38]. In accordance with other studies which indicate that n-3 PUFA is metabolized differently based on sex, we report significant differences by sex in linoleic and linolenic acid [20].

Although the functionality of UCP3 is thought to be similar to UCP1, upregulation is not always associated with mitochondrial uncoupling and/or thermogenesis. In effect, this mechanistic link may be a consequence of other UCP3-mediated functions, including lipid handling and prevention of damage due to reactive oxygen species [39,40]. Thus, obesity- or HFD-induced hepatic UCP3 expression would be expected, given the increase of FFA fluxed to the liver and hepatic mitochondrial dysfunction associated with NAFLD [41], suggesting that increased UCP3 expression in male mice may occur in an attempt to protect the liver from obesity-associated damage [42]. Clinical studies have reported that as n-3 PUFA supplementation increases, plasma AA concentration decreases [43]. Interestingly, we report no significant differences in hepatic linoleic acid concentrations among male or female groups, but a reduction in hepatic AA in the male KO-HF group compared to the WT-HF male group. Possibly of significance, n-6 PUFAs, specifically AA, have a higher affinity for UCPs compared to n-3 PUFAs [44], which could explain the reductions in hepatic AA in the male KO-HF group. Accordingly, UCP3 expression was significantly higher in the male KO-HF group, which could have contributed to the upregulated AMPK [45,46] found in this group. Although AMPK is involved in several pathways, paired up-regulation between uncoupling proteins and AMPK activity [12,46,47] may potentially explain the unexpected increases in AMPK in the male KO-HF group due to increases in the AMP/ATP ratio resulting from UCP3 activation. Consequently, given our recent reports, and others, we expect AMPK-driven decreases in FA synthesis, particularly indicators of hepatic de novo lipogenesis, including *Fasn* and *Acaca* [19]. Thus, upregulated UCP3 driven increases in AMPK could be responsible for the reduced *Fasn* and *Acaca* (near significance) expression reported in the male KO-HF group in our study through phosphorylation and inhibition of ACACA [48]. This relationship could explain the surprising effects on markers of FA synthesis in the male KO-HF group, despite the elevated hepatic TG in this group compared to all others. Moreover, increases in P-AMPK with EPA in the WT group in females corresponds to significant reductions in expression of *Fasn* and *Acaca* in the WT-EPA compared to the WT-HF group.

In the liver, UCP2 is thought to be mainly expressed in Kupffer cells under normal conditions and more active in hepatocytes with the accumulation of TG [49] as a result of increased lipid peroxidation [50]. Previous studies, however, have found no impact of UCP2 KO on the presence or progression of steatosis in the livers of diet-induced obese mice [51]. Therefore, the role of UCP2 in steatosis is controversial and may require cell-specific investigations [52]. Moreover, our findings vastly differ in males and females, with female results indicating an EPA-mediated increase in UCP2 in both WT and KO groups. While no other study has reported this in the liver, one study has reported similar increases in Ucp2 expression with EPA treatment in cardiomyocytes [53].

A limited number of studies have examined the role of UCP1 in female mice [11], and even fewer have evaluated a beneficial role for n-3 PUFA in diet-induced obese female mice [54]. Winn et al. reported no significant impact on body weight but significant increases in hepatic steatosis in female UCP1 KO mice fed a HF diet (45% kcal fat) and housed at 25 °C [11]. However, we report a significant increase in body weight and total fat mass [23] but no effect on hepatic TGs in UCP1 KO female mice. Accordingly, there were no significant differences in mRNA levels of proteins involved in lipogenesis, mitochondrial or peroxisomal oxidation and UCPs between the HF groups in the WT and UCP1 KO female mice, generally indicating negligible UCP1 KO-dependent changes in hepatic tissue of female mice. Given the lack of hepatic response to UCP1 KO, it is unsurprising that we report no significant differences in markers of lipogenesis and oxidation in UCP1 KO female mice receiving EPA. The exception to this is the significant increases in *Ucp2* and *Ucp3* with EPA seen in female UCP1 KO mice, which may support previous reports that fish oil-induced increases in hepatic *Ucp2* expression are independent of metabolic benefits [55]. Moreover, EPA mediated increases in *Ucp3* expression were not expected since there were no significant increases in FA oxidation, which is usually associated with increased *Ucp3* expression [39,40].

Given controversy on housing temperature and translation to humans [56,57], findings in the female WT group suggest hepatic benefits of EPA supplementation independent of body weight and adiposity [23] and in a thermoneutral environment. In agreement with our previous work in male mice housed at 22 °C [19], we report significant decreases in markers of lipogenesis, including *Fasn* and *Acaca*, as well as significant increases in markers of mitochondrial oxidation, including *Cpt1a* and *Cpt2* with EPA in the WT female mice. Additionally, we found significant increases in markers of peroxisomal oxidation, including Acox1, with EPA supplementation in the WT female mice. We did not see these hepatic benefits in the male WT group with EPA, suggesting that temperature impacts hepatic benefits of EPA in a sex-dependent manner.

Mitochondrial GPD2 (also named mGPDH) is a key component of the electron transport chain which functions in catalyzing the oxidation of glycerol-3-phosphate to dihydroxyacetone phosphate, which is accompanied by the transfer of two electrons [58]. Zheng et al. 2019 recently reported decreased hepatic *Gpd2* expression in steatosis, and accordingly, attenuation with GPD2 overexpression in ob/ob and diet-induced obese mice [59]. To our knowledge, we are the first to report increases in hepatic *Gpd2* gene expression with EPA supplementation. In parallel with hepatic TG data, we report significant increases in *Gpd2* with EPA in the male KO group and in the female WT group. The potential role of GPD2 in NAFLD development via decreased G3P-stimulated respiration, coupled with our findings warrants the further mechanistic investigation of EPA-mediated increases in *Gpd2*.

Lastly, there are a few limitations to the current study. First, the study design did not include a low-fat or chow diet group since the primary focus of our research was to examine mechanisms related to diet-induced obesity and inflammation [19]. Second, comparable to previous studies in our lab [15], we utilized 36 g/kg EPA (translatable to 10 g/day in humans), which exceeds the current n-3 PUFA recommendation of 1–4 g/day [18]. Recent studies in our lab have provided evidence for similar metabolic benefits when HF fed mice were supplemented with 18 g/kg EPA [60], indicating that a lower dose may be sufficient. However, results utilizing liver tissue from this feeding study are pending and warrant further investigation. Although, consistent amounts of EPA from previous studies in our lab [15] allow for temperature-dependent comparisons in C57Bl/6 mice with diet-induced obesity and have highlighted the importance of temperature in mediating the benefits of EPA supplementation. Studies remain to be conducted in livers from UCP1 KO male and female mice at ambient temperature to determine whether the effects we reported here are temperature-dependent. It is worth noting that in male mice, we have reported similar metabolic effects of fish oil in UCP1 KO models fed a HF diet rich in fish oil housed at ambient temperature (25 ± 2 °C) [29]. Third, mouse age at the introduction of the experimental diet may also be a factor for consideration. However, studies in our lab have previously utilized this feeding strategy to induce obesity and examine the impact of fish oil [15]. Others have also utilized this strategy in UCP1 KO mice [11]. Lastly, there are other UCP1 KO models that may be necessary to consider in order to objectively unravel the mechanistic targets of fish oil related to identifying targets to combat obesity and NAFLD [61].

## 5. Conclusions

In summary, our study is the first to report effects of HF or HF-enriched EPA diet in the livers of WT and UCP1 KO male and female mice housed in a thermoneutral environment. UCP1 KO exacerbated HF diet-induced liver steatosis in a thermoneutral environment, but only in the male mice.

In the males, supplementation with EPA prevented hepatic steatosis in the UCP1 KO mice independently of body weight. Our findings suggest that EPA may reduce hepatic TGs in the male mice by upregulating mitochondrial as well as peroxisomal β-oxidation. Alternatively, EPA may upregulate metabolic futile cycling involving glycerol-3-phosphate dehydrogenase.

In females, we report negligible impact of UCP1 KO in HF diet-induced steatosis and no significant impact with EPA supplementation in the KO group. Interestingly, our findings suggest a trending reduction in liver steatosis in the WT group in female mice housed in a thermoneutral environment. Our data suggests these potential EPA improvements may occur through downregulated lipogenesis and increases in mitochondrial and peroxisomal β-oxidation. As in the males, EPA may affect pathways related to glycerol-3-phosphate dehydrogenase –related futile cycles.

Overall, our data suggest sex-dependent effects of both UCP1 KO and HF-diet induced hepatic steatosis and rescue with EPA supplementation. Paired with our previous reports, temperature may impact hepatic effects of EPA supplementation.

## Figures and Tables

**Table 1:** Role of sex, genotype, and diet in livers of UCP1 KO mice.

		Main Effects			Interactions			
Variable	Statistic	Sex (S)	Genotype (G)	Diet (D)	S × G	S × D	G × D	S × G × D
Final Body Weight	*p* F (1, 96)	**<0.0001** 259.3	**<0.0001** 73.57	**<0.0001** 17.14	**<0.0001** 21.58	0.30 1.09	0.35 0.89	0.51 0.44
Liver Triglycerides	*p* F (1, 32)	**<0.0001** 40.96	**<0.0001** 46.23	**<0.0001** 68.59	**<0.0001** 35.23	**0.0002** 17.27	**0.0003** 16.01	**<0.0001** 38.00
P-AMPK/AMPK	*p* F (1, 24)	**0.003** 10.90	0.62 0.25	0.71 0.14	**<0.0001** 93.56	**0.0004** 16.98	**<0.0001** 40.96	**0.005** 9.78
FASN/TBP	*p* F (1, 26)	0.98 0.0006	**0.002** 12.30	0.18 1.86	**0.003** 10.47	**0.048** 4.27	**0.01** 7.85	**0.01** 6.88
Mitochondrial Uncoupling								
*Ucp2*	*p* F (1, 32)	**<0.0001** 89.90	0.98 0.0006	**<0.0001** 21.99	0.07 3.58	**<0.0001** 45.87	**0.004** 9.57	0.30 1.12
*Ucp3*	*p* F (1, 32)	**0.003** 10.19	**0.002** 11.77	0.09 3.11	0.05 4.08	**<0.0001** 42.02	**0.002** 11.79	0.07 3.52
FA Synthesis								
*Acaca*	*p* F (1, 32)	**<0.0001** 27.9	**0.0001** 27.18	**0.006** 8.59	**0.006** 8.87	**0.01** 2.88	**0.02** 6.41	0.78 0.09
*Fasn*	*p* F (1, 27)	**<0.0001** 34.25	**0.0002** 18.86	**<0.0001** 33.21	0.94 0.01	**0.009** 7.94	**0.004** 10.02	0.35 0.91
*Dgat2*	*p* F (1, 36)	**<0.0001** 58.66	**0.0003** 16.41	0.56 0.34	**0.02** 6.30	0.21 1.65	**0.0004** 15.55	**0.0004** 15.41
FA Oxidation								
*Pparα*	*p* F (1, 40)	**0.0003** 15.99	**0.04** 4.48	0.14 2.22	0.60 0.28	0.14 2.29	0.88 0.02	**0.009** 7.56
*Cpt1a*	*p* F (1, 36)	**0.0002** 16.67	**0.001** 12.62	**0.01** 6.79	**0.01** 7.40	0.96 0.002	0.46 0.55	**0.0004** 15.10
*Cpt2*	*p* F (1, 36)	**0.04** 4.41	**0.0004** 15.40	**<0.0001** 33.86	0.69 0.16	0.98 0.0004	0.44 0.60	**<0.0001** 29.17
*Acadvl*	*p* F (1, 36)	**0.02** 5.95	**0.03** 5.09	**<0.0001** 23.89	0.84 0.04	**0.03** 5.50	0.27 1.28	**0.007** 8.17
*Acat1*	*p* F (1, 36)	0.51 0.45	**0.002** 10.65	**0.03** 5.01	**0.0005** 14.90	0.67 0.18	0.80 0.06	0.08 3.21
*Ehhadh*	*p* F (1, 36)	0.79 0.07	**0.03** 5.68	**<0.0001** 42.84	**<0.0001** 23.72	0.20 1.73	0.19 1.77	**0.0009** 12.99
*Pex5*	*p* F (1, 36)	0.24 1.38	0.05 4.00	0.12 2.52	**0.02** 6.58	0.27 1.25	0.51 0.44	**0.01** 7.52
*Acox1*	*p* F (1, 36)	0.13 2.44	**0.01** 6.87	**<0.0001** 22.65	**0.008** 7.95	0.92 0.009	0.57 0.32	**0.02** 6.52
Futile Cycle								
*Gpd1*	*p* F (1, 36)	**<0.0001** 57.29	0.52 0.42	**0.01** 7.22	0.08 3.19	0.28 1.18	0.08 3.23	**0.0004** 15.42
*Gpd2*	*p* F (1, 36)	**<0.0001** 24.40	**<0.0001** 51.07	**<0.0001** 25.93	**0.006** 8.44	**0.002** 11.88	0.47 0.52	**<0.0001** 44.04

S, sex; G, genotype; D, diet; × indicates interactions, data are expressed as mean ± SEM, values bolded to highlight significance.

**Table 2:** Fatty acid composition (%) of liver tissue in male and female mice from each group.

		Group					Main Effects			Interactions			
Fatty acid	Sex	WT-HF	WT-EPA	KO-HF	KO-EPA		S	G	D	S × G	S × D	G × D	S × G × D
Palmitic acid 16:0	M	19.65 ± 0.91	19.14 ± 0.28	21.04 ± 0.34	20.08 ± 0.20	*p*	**<0.0001** 76.78	0.24 1.46	**0.008** 8.71	0.06 4.03	**<0.0001** 24.89	0.64 0.23	0.90 0.02
	F	21.80 ± 0.58 ^ac^	24.81 ± 0.42 ^b^	21.63 ± 0.52 ^c^	24.39 ± 0.30 ^ab^	F (1, 20)							
Palmitoleic acid 16:1 (n-7)	M	1.59 ± 0.41 ^a^	0.21 ± 0.21 ^b^	1.53 ± 0.21 ^a^	1.17 ± 0.22 ^ab^	*p*	**0.04** 4.85	0.40 0.73	**0.003** 11.79	0.25 1.41	0.60 0.28	0.13 2.43	0.46 0.57
	F	2.05 ± 0.45	1.23 ± 0.07	1.80 ± 0.26	1.34 ± 0.46	F (1, 20)							
n-6 PUFAs													
Linoleic acid 18:2 (n-6)	M	13.31 ± 0.76	13.36 ± 0.79	12.24 ± 0.59	12.52 ± 0.50	*p*	**<0.0001** 1012	0.25 1.38	0.88 0.02	0.24 1.47	0.58 0.31	0.86 0.03	0.91 0.01
	F	0.33 ± 0.09	0.02 ± 0.02	0.32 ± 0.08	0.06 ± 0.03	F (1, 20)							
Arachidonic acid 20:4 (n-6)	M	7.18 ± 1.21 ^a^	4.14 ± 0.39 ^ab^	4.18 ± 0.43 ^ab^	3.10 ± 0.21 ^b^	*p*	0.50 0.48	**0.03** 5.42	**0.0001** 22.77	0.23 1.51	0.27 1.30	0.20 1.78	0.70 0.16
	F	7.29 ± 0.84	3.42 ± 0.41	6.14 ± 1.60	3.33 ± 0.42	F (1, 20)							
n-3 PUFAs													
α-Linolenic acid 18:3 (n-3)	M	0.06 ± 0.06	0.00 ± 0.00	0.13 ± 0.04	0.00 ± 0.00	*p*	**<0.0001** 147.4	0.80 0.07	0.25 1.42	0.16 2.17	0.05 4.35	0.83 0.05	0.33 0.99
	F	0.41 ± 0.05	0.41 ± 0.03	0.33 ± 0.05	0.38 ± 0.04	F (1, 20)							
Eicosapentaenoic acid 20:5 (n-3)	M	0.00 ± 0.00 ^a^	6.78 ± 0.73 ^b^	0.00 ± 0.00 ^a^	6.18 ± 0.35 ^b^	*p*	0.28 1.22	0.11 2.82	**<0.0001** 329.5	0.39 0.79	0.28 1.22	0.11 2.82	0.39 0.79
	F	0.00 ± 0.00 ^a^	8.29 ± 0.45 ^b^	0.00 ± 0.00 ^a^	6.34 ± 1.33 ^b^	F (1, 20)							
Docosahexaenoic acid 22:5 (n-3)	M	2.27 ± 0.60 ^a^	8.35 ± 0.50 ^b^	1.49 ± 0.18 ^a^	6.86 ± 0.28 ^b^	*p*	0.89 0.02	0.14 2.42	**<0.0001** 116.6	0.36 0.089	0.08 3.30	0.97 0.001	0.43 0.65
	F	3.10 ± 0.34 ^ab^	6.80 ± 0.69 ^b^	2.44 ± 0.58 ^a^	6.90 ± 1.54 ^b^	F (1, 20)							

S, sex; G, genotype; D, diet; × indicates interactions, data are expressed as mean ± SEM, *n* = 4, means without a common letter (a, b, c) differ in each male or female group; means with any common letter are not different from each other (a or b vs. ab; a or c vs. ac); values bolded to highlight significance.

## Data Availability

Data supporting reported results can be obtained from corresponding author, upon reasonable request, following publication.

## Supplementary Materials

The following are available online at https://www.mdpi.com/article/10.3390/biomedicines9111549/s1, Table S1: Diet Composition; Table S2: Primer sequences used for qPCR.
