# Supplementary material for: Sex-Dependent Effects of Eicosapentaenoic Acid on Hepatic Steatosis in UCP1 Knockout Mice
Source: Biomedicines. 2021 Oct 27;9(11):1549. doi: 10.3390/biomedicines9111549 (PMC8615653; doi:10.3390/biomedicines9111549)
Supplement: Supplementary file 1 [file biomedicines-09-01549-s001.zip › biomedicines-1395751-supplementary.pdf]

Supplementary Material

Table S1. Diet Composition, D08112003.

Dietary Component	HF-EPA		HF	
	**gm**	**kcal (%)**	**gm**	**kcal (%)**
Protein	24	20	24	20
Carbohydrate	41	35	41	35
Fat	24	45	24	45
Total	100		100	
	**gm**	**kcal**	**gm**	**kcal**
Casein	200	800	200	800
L-Cystine	3	12	3	12
Corn Starch	72.8	291	72.8	291
Maltodextrin 10	100	400	100	400
Sucrose	172.8	691	172.8	691
Cellulose	50	0	50	0
Soybean Oil	25	225	25	225
Lard	139.5	1256	177.5	1598
AlaskOmega EE (820 mg EPA/g)	38	32	0	0
Mineral Mix S10026	10	0	10	0
Di Calcium Phosphate	13	0	13	0
Calcium Carbonate	5.5	0	5.5	0
Potassium Citrate, 1 H2O	16.5	0	16.5	0
Vitamin Mix V10001	10	40	10	40
Choline Bitartrate	2	0	2	0
Vitamin E Acetate, 50% (500IU/gm)	0.13	0	0.13	0
FD&C Yellow Dye #5	0.05	0	0	0
FD&C Red Dye #40	0	0	0.025	0
FD&C Blue Dye #1	0	0	0.025	0
Total	858.27	4057	858.28	4057
EPA content in the diet (g/kg diet)	36		0	

Table S2. Primer sequences used for qPCR.

Gene	Forward	Reverse
Ucp2	CCAGAGAAGAGCACCAGTTC	GGACCTAACACAATGACCAGAT
Ucp3	GATGTGGTGAAGGTCCGATT	CTGGCGATGGTTCTGTAGG
Acaca	GCAGCAGTTACACCACATACA	CATTACCTCAATCTCAGCATAGCA
Fasn	TGTATCCTGCTGTCCAACCT	GGCTTGTCCTGCTCTAACTG
Dgat2	CCTCATCGCCGCCTACTC	GAGCCAGGTGACAGAGAAGAT
Pparα	ATCCACGAAGCCTACCTGAA	AATCGGACCTCTGCCTCTT
Cpt1a	GAGACAGACACCATCCAACAC	GAGCCAGACCTTGAAGTAACG
Cpt2	CAGCACAGCATCGTACCCA	TCCCAATGCCGTTCTCAAAAT
Acadvl	TGGCTCGGATGGCTATTCT	TGCTGATGGCGGCTTCTA
Acat1	TCACGGCAGAAGCAGGAT	GCACAATCTCAGCACGGAAG
Ehhadh	TCATTCCTAGCCGATACTCTTCC	GTTACCAACAACGACTCCAATCT
Pex5	CAGGCAGAGTTGGAGGAGAT	CTTGTCATAGGAAGCGGATGTG
Acox1	CGCCACCTTCAATCCAGAG	TTCTTAACAGCCACCTCGTAAC
Gpd1	CGGAGACAAGCGGAAGGA	CCAGAGGACAGCAAGGAAGT
Gpd2	TGAGTGGCAAGTTGGATATAATGG	GCTACACGCTACAAGATTCAGAA
